# Stretching the Boundaries: Tanzanian Pharmacy Workers’ Views and Experiences of Providing STI Services for Men Who Have Sex with Men

**DOI:** 10.1371/journal.pone.0166019

**Published:** 2016-11-03

**Authors:** Markus Larsson, Karen Odberg Pettersson, John Kashiha, Michael W. Ross, Anette Agardh

**Affiliations:** 1 Division of Social Medicine and Global Health, Department of Clinical Sciences Malmö, Lund University, Malmö, Sweden; 2 Community Health Education Services & Advocacy (CHESA), Dar es Salaam, Tanzania; 3 Programme in Human Sexuality, Department of Family Medicine and Community Health, Medical School, University of Minnesota, Minneapolis, Minnesota, United States of America; University of Toronto, CANADA

## Abstract

**Objective:**

To explore the views and experiences of providing assistance and treatment of sexually transmitted infections to same-sex practicing male clients among service providers at pharmacies and drugstores in Dar es Salaam, Tanzania. Previous research suggests that sexually transmitted infections are an increasing concern for this population. Due to stigma and discrimination, men who have sex with men face limited access to treatment, which might contribute to increased self-medication. However, limited research has been conducted on the role of the pharmaceutical service provider with regards to this population in sub-Saharan Africa.

**Method:**

In January 2016, 16 service providers at private pharmacies and drugstores with previous experience of providing services to this population were purposively selected for open-ended face-to-face interviews. The analysis was guided by the grounded theory approach.

**Results:**

The process that emerged was labelled “Stretching Boundaries for Pharmaceutical Responsibilities”. This reflected informants’ perceptions of themselves as being involved in a transition from having limited engagement in the care of same-sex practicing male clients to becoming regular service-providers to this group. Findings further revealed that the emotional commitment they developed for clients through this process led to a transgression of provider-client boundaries, which undermined objective decision-making when clients lacked prescription. Financial interests also emerged as an underlying motivation for providing incomplete or inaccurate drug dosages.

**Conclusions:**

Further studies are required to better address incentives related to unregulated sale of drugs. Inter-professional networks between pharmacy and healthcare workers could support the development of targeted treatment for men who have sex with men and other key populations.

## Introduction

Evidence suggests that sexually transmitted infections (STIs) constitute a growing problem for men who have sex with men (MSM) in Tanzania. In 2014, Ross and colleagues reported in a respondent-driven survey of 300 MSM that 21.4% in Dar es Salaam and 4.4% in the smaller city of Tanga had tested positive for gonorrhoea and/or chlamydia [1]. In Dar es Salaam 2.5% had tested positive for syphilis and in Tanga 8% for hepatitis B (HBV). Another study conducted in Dar es Salaam found that prevalence of herpes simplex virus type 2 (HSV 2) was 40.9%, syphilis 0.11% and HBV 0.5% among the 753 MSM surveyed [2]. In addition, HSV 2 infection was found to be associated with HIV infection.

Perceived and actual stigma may play a role in obstructing healthcare access for MSM who experience STI problems [3, 4]. In our previous qualitative study from Dar es Salam, narratives revealed that participants’ experiences of discriminative actions by healthcare workers due to their sexual orientation and behaviours discouraged them from seeking healthcare services [4]. This is in line with the findings from a quantitative study in Dar es Salaam, which demonstrated that 14.8% of 200 surveyed MSM perceived stigma as an impediment to HIV services [5].

Self-treatment or self-care, i.e. when a person is self-medicating with modern pharmaceutical drugs [6], occurs to a higher extent when available healthcare alternatives are limited, expensive, and of poor quality [7]. Self-treatment may, however, also occur as a reaction to stigma and discrimination. Our previous findings showed that MSM in Tanzania preferred obtaining drugs directly from the pharmacy or drugstore since they not were required to provide any explanation for their conditions, which ensured their privacy [4]. Fear of stigma in healthcare was also identified as one of the reasons for self-treatment in another qualitative study concerning MSM in Dar es Salaam by Magesa and colleagues [3]. Similar findings have been reported across the sub-Saharan African continent. In Nigeria, a report revealed that 25% of those MSM with a STI symptom had approached a pharmacy for advice and treatment [8]. Self-treatment among MSM has also been documented in qualitative studies from Senegal and Uganda [9, 10]. However, the unregulated use of antimicrobial agents could contribute to the development of drug resistance, which hampers effective treatment and care [11].

Very few, if any, studies in Tanzania have attempted to understand the perspectives of those working at pharmacies and drugstores when it comes to providing services to MSM. Given the available evidence regarding their perceived role by MSM as service providers [4, 8, 9], it is likely that pharmacists and drugstore staff exert considerable influence on the health of MSM clients. In light of the high levels of STIs in the MSM population, and the accessibility of pharmacies and drugstores, increased knowledge concerning these service providers’ experiences of interacting with MSM clients is critical. This knowledge could inform future initiatives that target the sexual health of MSM. Thus, the purpose of this study was to explore the views and experiences of providing STI treatment and assistance to MSM among service providers at pharmacies and drugstores in Dar es Salaam.

## Methods

### Study setting

The present study was conducted at private pharmacies and drugstores in Dar es Salaam, Tanzania. Dar es Salaam has according to the latest census a population of over four million [12]. Sexual acts between two males are prohibited according to the Tanzanian penal code [13]. Although it remains uncertain whether the law has been implemented recently, MSM face problems related to stigma and violence in their communities. A study conducted among 200 MSM in Dar es Salaam showed that they were subjected to verbal abuse (48.5%) and physical violence (29.5%)–mainly from people in the street–due to their sexual orientation and behaviours [14].

Pharmaceutical regulation in Tanzania is under the responsibility of the Tanzania Food and Drugs Authority (TFDA) according to the Food, Drugs and Cosmetics Act from 2003 [15]. The authority oversees manufacture, import, export, and retail of drugs in the country. There are two types of drug-specific retailers in the private sector: Part I and Part II pharmacies [16]. The first type is operated by a pharmacist and is licensed to sell both medicines that require a prescription as well as those sold over the counter [17]. In order to obtain a licence, the pharmacist is required to register with the TFDA to get approval upon inspection of premises [15]. The registration must be renewed annually. In 2010, 352 Part I pharmacies were registered in Tanzania [18].

Part II pharmacies, *Duka la Dawa Baridi* in Swahili or drugstores, are only authorised to sell a limited list of over-the-counter (OCT) medicines, i.e. non-prescription medicines, such as analgesics [19]. They too need to obtain a licence from TFDA, which should be renewed annually [16]. Although the store owners are not required to possess specific formal qualifications, staff interacting with customers should have basic medical knowledge (i.e. nurse, nurse assistant, pharmacy assistant) [16]. Part II pharmacies are much more available than Part I pharmacies. More than 6000 drugstores were operating in 2010, but the actual figure may be considerably higher as some stores may not be officially registered [18]. Since between 60–70% of all Part I pharmacies are located in Dar es Salaam, people outside urban areas have relied on Part II pharmacies [20].

Due to problems related to the enforcement of regulations, Part II pharmacies have illegally stocked and provided prescription-only drugs [17]. The TFDA therefore initiated the accredited drug dispensing outlet (ADDO) programme in 2003 to capacitate personnel at Part II pharmacies in the area of essential drug provision [21, 22]. Accredited Part II pharmacies are locally called *Duka la Dawa Muhimu* and are allowed to dispense some prescription-only drugs such as antimicrobials [22]. In 2013 there were approximately 9000 accredited stores spread across the country [22].

### Informants

This study was approved by the Senate Research and Publication Committee at Muhimbili University of Health and Allied Sciences, Tanzania (ref. no. 2015-11/AEC/Vol.IX/102). Sixteen pharmacy and drugstore workers were recruited in early 2016 using purposive and snowball sampling (see Table 1 for informants’ characteristics). Recruitment was facilitated by a local community-based organisation working with key populations and gender and sexual minorities populations. Since the purpose was to elicit informants’ views and experiences of providing services to MSM, it was considered important to meet with those who possessed previous experiences of assisting MSM clients. It was therefore decided that the organisation as a first step should conduct an inventory of the pharmacies and drugstores utilised by their members. As a second step, the first and the last authors (ML and AA), in consultation with the community-based organisation, selected informants based on a) ability to provide privacy for the interview and b) geographical distribution of the workplace. This process continued as the interviews progressed.

**Table 1:** Informant characteristics.

Informant	Sex	Professional background	Place of work
1	Female	Nurse (owner)	Drug store
2	Female	Pharmacy assistant	Pharmacy
3	Female	Nurse	Drug store
4	Female	Pharmacy assistant	Pharmacy
5	Male	Pharmacist	Pharmacy
6	Male	Pharmacist technician	Pharmacy
7	Female	Nurse	Pharmacy
8	Male	Teacher (owner)	Drug store
9	Female	Nurse	Drug store
10	Female	Nurse assistant (owner)	Drug store
11	Female	Medical doctor (owner)	Drug store
12	Female	Nurse (owner)	Drug store
13	Female	Pharmacist	Pharmacy
14	Female	Medical assistant (owner)	Drug store
15	Female	Nurse	Pharmacy
16	Female	Pharmacist (owner)	Pharmacy

Finally, to be eligible for the study, informants had to work at a pharmacy or drugstore in Dar es Salaam, have had previous contacts with MSM health-seeking clients, be available for an interview during the time of data collection, and be over the age of 18 years. Since the informants worked at both pharmacies and drugstores (i.e. Part I and Part II pharmacies), they are as a group referred to as *pharmacy workers* in this study.

### Study Procedures and Instruments

All interviews were audio-recorded and took place in a separate section at the informants’ workplace with no one else present, or at a locality nearby- depending on the informant’s preference. Before the interview began, we requested permission to record the interview and made it clear that in order to protect their anonymity, no information would be recorded that could identify the informants. All pharmacy workers in the study agreed to have their interview recorded. Informed verbal consent was obtained and recorded from all individual participants included in the study by reading the letter of consent at the beginning of the interview. Pharmacy workers were informed that participation was anonymous, voluntary, and that they could refuse participation or withdraw from the interview at any time. All persons who were approached and who fulfilled the criteria agreed to participate.

In order to pursue in-depth information about pharmacy workers’ views and experiences of providing STI services to MSM clients, the interviewers (ML, AA) entered each interview situation with an open approach [23]. A typical example of a question that was used is the following: “Could you discuss your experiences of providing services to MSM clients?”. An interview guide had been developed, which covered topics that were to be explored. These included, for example, experiences and practices around syndromic management, what types of problems MSM clients approached them with, and challenges related to providing services to MSM clients. Prior to the interviews, these topics had been pilot tested for the purpose of clarity and appropriateness on members from the community-based organisation. The interview guide was developed in English and translated into Swahili (the official language in Tanzania) by representatives from the community-based organisation (S1 and S2 Files). The interviews ranged between 40 minutes and 1.5 hours, and were conducted in English. Informants were always given the opportunity of having an interpreter present if they felt the need for it. In those cases, a representative from the community-based organisation assisted and translated the questions to the informant, who could answer in Swahili (four interviews, among which, in two interviews only for some of the responses). No financial incentive was provided to the informants. The interviews were transcribed verbatim after each session. The same interpreter who had participated in the interviews translated the passages into English that had been provided in Swahili.

### Data Analysis

The analysis was guided by the grounded theory approach as described by Strauss and Corbin [24, 25]. At the end of an interview, ML and AA wrote reflective notes to summarise impressions and highlight potential concepts. This facilitated comparison between already collected data and new data in accordance with the constant comparison method [24]. A preliminary analysis was conducted after a few interviews, which provided the authors (ML, AA) with the opportunity to discuss recurring categories but also to share findings with representatives from the community-based organisation to receive their feedback on the data interpretations.

**Fig 1:**
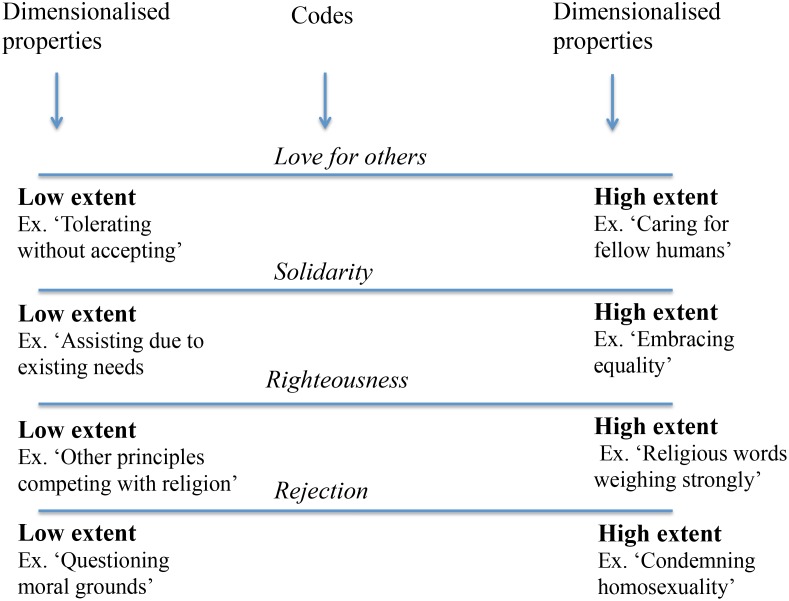
An example of properties and dimensions of the sub-category ‘Searching for answers in religion’. The properties of the codes ‘love for others’, ‘solidarity’, ‘righteousness’ and ‘rejection’ have dimensions of low extent properties and high extent properties as illustrated in the figure.

Open coding, step 1, was conducted using MAXQDA12 (VERBI Software, Berlin, Germany), a qualitative data analysis software package recommended by Strauss and Corbin [24]. Each transcribed interview was reviewed line-by-line and contents were conceptually labelled with a code. As the interviews progressed, more codes were developed and compared with each other. Similar concepts were merged together and primary categories that described their contents were formulated [24]. Memos were written to help make sense of the properties (characteristics or attributes) and dimensions (location along a continuum) of codes that formed different categories and sub-categories (see Fig 1 for an example of properties and dimensions of a sub-category). As a second step, categories were related with each other through axial coding [24]. To understand their relationship, and to identify possible sub-categories, Strauss and Corbin’s “coding paradigm” was used; thus the software package was not applied for axial and selective coding [25]. This aided the process of making sense of the conceptual linkages by outlining conditions, context, strategies, and consequences. The third and last step was selective coding [24], i.e. conceptualisation of the core category, “*stretching the boundaries for pharmaceutical responsibilities*”. The data, i.e. all interviews and analytical memos, were re-examined, applying the constant comparison technique [24] in order to identify connections between the core category and the other categories. This analysis was conducted by ML in collaboration with KOP and AA.

After 16 interviews there was a common understanding that the views and opinions of pharmacy workers had been sufficiently covered to enable the identification of a potential model that would describe pharmacy workers’ experience of service provision to MSM clients.

## Findings

### Stretching Boundaries for Pharmaceutical Responsibilities

**Fig 2:**
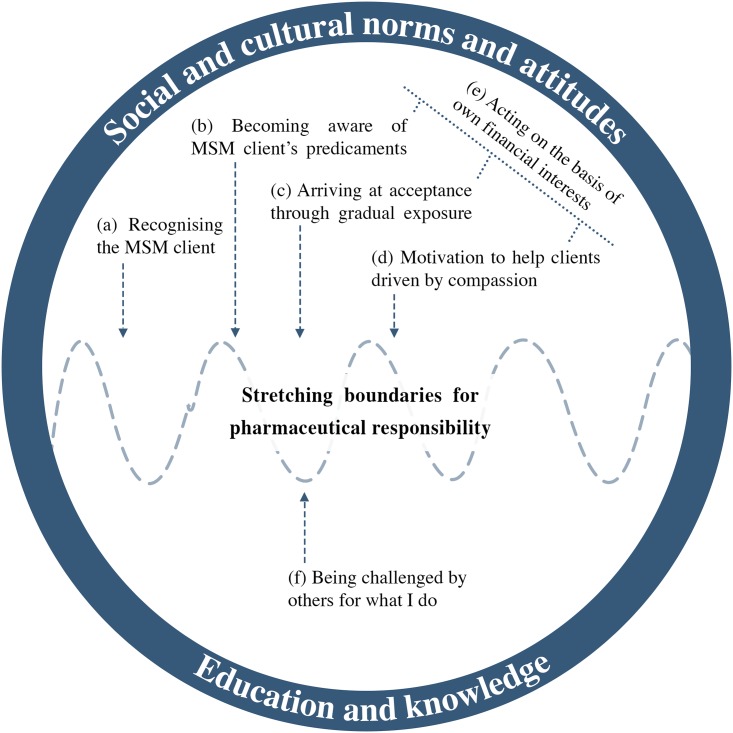
Conceptual model describing pharmacy workers' views and experiences of providing STI services for MSM clients. The core category "Stretching boundaries for pharmaceutical responsibilities" represents the transition from having limited engagement to becoming regular care providers. The categories (a-f) show the views and experiences involved in this process, and the contextual framework (illustrated by the blue outer rim) shows the influence of key external factors, i.e. socio-cultural norms concerning homosexuality and societal levels of knowledge and understanding.

The analysis of 16 individual in-depth interviews provided rich descriptions of pharmacy workers’ experiences of assisting MSM clients. The model that emerged illustrates that informants perceived themselves to be involved in a transition from having limited engagement in MSM care to becoming regular service-providers to MSM clients through a process labelled “*Stretching Boundaries for Pharmaceutical Responsibilities”* (Fig 2). This core category consisted of six categories describing informants’ experiences related to the provision of services to MSM clients (Table 2). The categories were identified through grouping similar sub-categories together (Table 2).

**Table 2:** Categories and sub-categories.

Sub-category	Category
Existing conceptions of same-sex sexuality Trusting intuitions about clients’ sexuality Questioning possibility to recognise a MSM client	Recognising the MSM client
Specific incidents making it impossible to close one’s eyes Avoiding unnecessary exposure “Fishing around”	Becoming aware of MSM clients’ predicaments
Increased understanding through interaction Searching for answers in religion Prioritising role as service-provider	Arriving at acceptance through gradual exposure
Sympathising with clients’ fears Struggling to gain necessary trust Motivating and comforting Making extra efforts to display confidentiality Building abiding relations “Treating him according to the situation” Insisting on prescription	Motivation to help clients driven by compassion
Providing drugs only according to payment Financial incentives superseding need for prescription Establishing a good reputation in MSM community	Acting on the basis of own financial interests
Colleagues objecting to assist MSM clients Community reacting against me Coping with accusations	Being challenged by others for what I do

The phenomenon “Stretching Boundaries for Pharmaceutical Responsibilities” describes the dynamic process that emerged during the course of pharmacy workers’ interaction with MSM, as the relationships created enabled informants to manage and challenge own concerns and insecurity. While these relationships varied in their extent and depth, they were perceived as central for the delivery, and quality, of services to MSM clients. Financial interests appeared as a critical issue for pharmacy workers, who stressed their own role as businessmen and women. This process, which included identification of financial gains and prevention of loss, influenced perceptions of services provided. Furthermore, contextual factors such as cultural norms related to homosexuality and societal level of knowledge and understanding also influenced informants in the process of providing services to MSM clients (outside circle in Fig 2).

The findings are presented below and structured according to the conceptual model (Fig 2): categories are in bold and sub-categories italicized and underlined within single quotation marks.

### Recognising the MSM client

Opinions and images of homosexuality permeated perceptions of MSM clients. These were embedded in interpretations of gender roles and gender expressions, which had created a stereotype of MSM clients as effeminate. These preconceived ideas were used to distinguish MSM clients and revealed the interplay between socio-cultural norms, and knowledge and education about homosexuality.

*‘Existing conceptions of homosexuality’* seemed to govern how informants interpreted signs of someone’s sexual orientation. Feminine traits, i.e. “*the way they dress and behave*” (Informant 8, male), as interpreted by informants, dominated explanations of how they spotted a MSM client:

*“Many MSM are gay—that is why they look very feminine*.*”*

(Informant 1, female)

These associations were central features in the interviews and appeared to constitute a benchmark against which informants recognised MSM clients. Clients with non-conforming gender role expressions, and who displayed these explicitly, appeared to challenge pharmacy workers:

*“But of course there are those who are calling for attention*. *I mean if you have make-up everyone will know you are gay!”*

(Informant 6, male)

Informants also drew conclusions about MSM clients based on more loosely defined characteristics. They were *‘trusting intuitions about clients’ sexuality’*, which also relied on manners, personality, and intonation:

*“You know it is more the way they behave*. *They can talk with me in a funny voice or just be so shy*. *So I just recognise how they act…that is how I do it*.*”*

(Informant 3, female)

Informants believed that clients’ own awareness of their sexual orientation affected their behaviour as customers:

*You know…it is something you reveal about yourself…that you are doing something different from others…so you feel shy*.*”*

(Informant 7, female)

However, informants were also ‘*questioning possibility to recognise a MSM client’* and stressed the importance of letting the client decide whether he wanted to introduce sexuality into the consultation or not:

*“You cannot recognise somebody that he is a MSM until he tells you*. *You cannot identify otherwise*. *Maybe some you can see because they are like women with kanga* [traditional dress, author’s remark] *or long hair*. *But others you cannot tell*.*”*

(Informant 11, female)

### Becoming aware of MSM clients’ predicaments

Recognising MSM clients’ challenges in obtaining pharmaceutical services seemed to be an important step for approaching clients. This included events and situations, which triggered informants’ minds and afforded them with a context to interpret clients’ behaviours. The awareness provided a foundation for continued engagement in MSM clients.

Pharmacy workers gave vivid details of their encounters with MSM clients. When they explained how their engagement with these clients started, it appeared that ‘*specific incidents making it impossible to close one’s eyes’* constituted an important element:

*“One MSM, who came here to access medication, it is a very sad story, told me about his experiences of previous pharmacy workers, who had mistreated him*. *They had pointed fingers at him after he had been telling them ‘I have done this and I have that’, ‘I have had unsafe sex and I have problems down at my private parts’*. *When he was passing close to the shop they were pointing fingers at him and he was just feeling dead inside*. *He said ‘I was feeling so bad when I went to the other pharmacies because some people were stigmatising me´*.*”*

(Informant 14, female)

Understanding MSM clients’ challenges could also occur through the observation of certain health-seeking behaviours that seemed unique to this group of clients. Informants had noticed that clients took various measures to ‘*avoiding unnecessary exposure’*. This was believed to be a consequence of previous exposure to gossip and discrimination:

*“They do not want to walk around and be seen in daytime*. *They do not want finger points from others*. *That is why they come late in the evening hours*.*”*

(Informant 1, female)

Another unique behaviour that attracted informants’ attention was when clients drifted away from what was believed to be their original reason for coming to the dispensary. Informants claimed that this behaviour, tantamount to ‘*fishing around*’ (Informant 8, male), was deployed as a strategy to avoid unnecessary exposure of clients’ sexual orientation or behaviours in certain situations:

*“They are feeling too shy to talk about themselves and about homosexuality when they come here*. *If they find some other people in here they can ask some different questions*: *‘Do you have soap*?*’*, *‘Do you have sugar*?*’*. *Things that we do not even have in the pharmacy*!*”*

(Informant 15, female)

### Arriving at acceptance through gradual exposure

Managing one’s attitudes, views, and opinions of what was perceived as different or strange was understood as central to be able to engage in services and care for MSM clients. Different factors accounted for acceptability of clients’ behaviours and ultimately coming to terms with these. Acceptance was influenced by personal insights but also by religious beliefs. Moreover, it seemed that there was a distinction between acceptance based on humanitarian values and a more pragmatic one, akin to a professional tolerance.

Informants were candid in discussing how they had come to accept MSM clients. ‘*Increased understanding through interaction’* was frequently mentioned. These interactions seemed important for informants in order to make sense of the clients:

*“MSM clients have made it possible for me to know much more about them*. *Because before I did not know there was a group like that, that existed*. *But the more I interacted with them, the more I understood about the group*.*”*

(Informant 16, female)

*‘Searching for answers in religion’* revealed contradictions between informants’ beliefs and how these were translated into practice. Although beliefs sometimes clashed with the issue of homosexuality, informants shared how they had impacted on their perceptions of MSM clients:

*“For the first time it was very hard because I have two male kids*. *But for the time being I am very supportive of them* [MSM, author’s remark]. *I feel that they are human beings like everyone else because of God*. *He made me realise that everyone is a human being*.*”*

(Informant 2, female)

However, religious beliefs also created dilemmas about how to handle MSM clients, as beliefs sometimes did not accommodate homosexuality:

*“I feel homosexuality is very dangerous, especially for men and sometimes they get into problems*. *It is too dangerous*. *My religion does not allow it*. *So it complicates for me*. *But I try*. *I really do*.*”*

(Informant 15, female)

This statement revealed the inner conflict informants struggled with, as religion played an important role in their lives. However, accounts also showed that they were able to distance themselves from religious beliefs. They pondered where to draw the line between faith and practice and discussed the importance of being able to balance one’s own personal beliefs.

*“I do not see any problems around the religious perspective when it comes to MSM*. *Christianity is just about your beliefs*. *It is about your own connection with God*. *I do not see the logic of chasing other people when we are all the same*.*”*

(Informant 16, female)

*‘Prioritising role as service-provider’* was a crucial aspect for informants. This role, they said, became critical once they started to engage with MSM clients, as it helped them to make sense of why they should assist these persons:

*“It was hard for me to meet MSM clients but I accepted it*. *It is my role*. *My role as a pharmacy worker*. *To help people*. *So that is how it started*.*”*

(Informant 1, female)

Pharmacy workers had an expectation that professional integrity was superior to personal attitudes. The following quote revealed that a tolerance based on professional principles concerning service-provision rather than on acceptance had emerged:

*“I do not stigmatise or discriminate MSM but I feel, to be honest, bad by that kind of behaviour…same-sex…but because of my work I am supposed to give everyone treatment*.*”*

(Informant 3, female)

### Motivation to help clients driven by compassion

Emotional involvement in clients emerged as a central feature and it seemed that concerns for clients constituted an important motivation for service provision. The belief that clients generally lacked incentives to confide in pharmacy workers had generated a conviction that certain actions and procedures were required to restore the trust. Personalised ties had emerged, which challenged views of MSM clients as merely like any other client, and they were perceived as ‘friends’ or ‘sons’. Furthermore, emotional involvement in clients tended to influence professional ethics when decisions were to be taken regarding the provision of STI related drugs.

*‘Sympathising with clients’ fears* was an evident feature in pharmacy workers’ accounts:

“*I feel sorry for them*. *They look so uncomfortable inside the store and if there are many people in here I can see that they walk away*. *So then they do not come in*.*”*

(Informant 4, female)

The importance of trust was frequently mentioned by informants, and pharmacy workers had identified certain actions to ensure that this was created during interaction with clients as they were *‘struggling to gain necessary trust’*.

*“They are not straightforward*. *They are hesitant to speak out so you have to take time to talk with them, to assure them that it is a secret*. *I tell them ‘If you tell me the truth I am here for you and I can help you to be well, so you just have to be open and do not worry’*.*”*

(Informant 1, female)

As shown by the statement, informants also described displaying non-judgemental attitudes to clients and bringing counselling skills into the interaction. Pharmacy workers explained that ‘*motivating and comforting’* gradually became an integrated part of their work. They spent time on trying to make clients “*accept their situation and explain treatment options*” (Informant 3, female). The following quote describes how informants dealt with clients who displayed frustration and despair:

*“They are getting angry because they have given up*, *and think that they are not going to get well with their situation*. *So that makes them frustrated and mad*. *But I counsel them and try to motivate them and advise them to go to the correct healthcare provider or centre*.*”*

(Informant 16, female)

Informants discussed the trust issue vividly, and maintaining trust seemed to be challenging due to clients’ fear of exposure of their sexual orientation and behaviours. ‘*Making extra efforts to display confidentiality’* was therefore regarded as essential in order to uphold their relational trust with clients:

*“I assure them confidentiality and I actually tell them that ‘Even when I am sitting outside here and talking to other people, do not think that I am talking about you*. *Come any time and ask me anything’*.*“*

(Informant 16, female)

The value of *‘**building abiding relations’* was expressed frequently by pharmacy workers, and clients were referred to as friends. These relations had created a foundation for the delivery of services to MSM clients:

“*For me*, *the way they approach me*, *they are open about their sexual situations*. *They usually tell me openly*: *‘I’m practicing anal sex’*, *or ‘I’m doing same-sex partnerships’*. *So they are quite open to me*. *I think it is because* [they, author’s remark] *see me as a friend*.*”*

(Informant 12, female)

Relations with clients were also described in affectionate terms, which indicated aspects of caretaking and supporting:

“*Now I feel like I am a mother to them*.*”*

(Informant 7, female)

Pharmacy workers invested in their relationships with clients and sought to maintain durability and continuity in interactions, which is demonstrated by the following quote:

“*What I do is that when a client gets the medication and he goes home*, *I ask him to return back to give me feedback after using the medication*. *If I am not available I ask them to leave a message for me ‘Just tell the other lady that I have used this and that and it works like this’*. *Many have thanked me when they return back*.”

(Informant 15, female)

“*Treat him according to the situation*” is an in-vivo code (i.e. informant’s own words) and described informants’ attitudes to drug provision in situations when clients lacked prescription:

*“There are some who asks for treatment*. *After explaining what he suffers from I treat him according to the situation*. *But I always ask him to go to the doctor first and come back with a prescription*. *‘Then I know what to give you’*. *But if he comes without a prescription and it is serious, and I see this and know that the kind of disease is rare or like syphilis, I help him with treatment- even without a prescription*.*”*

(Informant 8, male)

As exemplified by the above statement, a common impetus for helping was the understanding of clients’ situations. It appeared that the ability to grasp clients’ suffering was offered as an explanation for expediting drugs without a prescription:

*“The aim is to help the person, so if you ask him to go to the doctor and come back with a prescription maybe he will not come back again*. *Because he does not want to go to the doctor or hospital due to stigma and discrimination*. *Since the aim is to help him you have to do this without asking him to go back for a prescription*.*”*

(Informant 1, female)

However, helping clients were also seen as ‘*insisting on prescription’* due to the problems unauthorised drug-provision could result in:

*“Sometimes you give them but sometimes you say ‘I do not want to give the drugs to you because I do not know if you know how to use them’*. *Then they leave and try somewhere else*. *That is why we have this problem with drug-resistance here in Tanzania*.*”*

(Informant 5, male)

### Acting on the basis of own financial interests

Financial gains emerged as an important motivational issue for provision of drugs to clients. This could result in a conflict arising between the role as service provider on the one hand and the role as businessman or woman on the other. While having a desire to assist those clients in need, pharmacy workers raised concerns about losing incomes when confronted with poverty-stricken clients. Faced with this dilemma, pharmacy workers seemed to arrive at a compromise when it came to drug provision for STI related conditions, which revealed a lack of commitment to STI protocols and STI treatment regimens and dosages. Furthermore, as service providers to MSM clients, they were aware of certain traits, or qualities, that were particularly appreciated by the MSM community and made efforts to emphasise them.

*‘Providing drugs only according to payment’* was a frequently recurring phenomenon expressed during the interviews. Financial constraints constituted barriers for clients to purchase full dosages of required drug regimens. Pharmacy workers emphasised that while they “*ran a business*” (Informant 13, female) and could not provide free drugs, they wanted to be able to provide at least a minimum level of help.

*“When they come they have a small amount of money and there is nothing you can do*. *You cannot give them three antibiotics if they come with 3000* [Tanzanian Schilling, author’s remark]. *At least you can give them some but it is not the exact dose*.*”*

(Informant 11, female)

They referred to commercial interests when justifying their actions:

*“Most of my clients can only afford to take one or two tablets*. *But it is not sustainable for my business to give them more tablets than they can pay for*.*”*

(Informant 13, female)

The accounts of pharmacy workers revealed that financial aspects resulted in insufficient dosages, thus compromising standards regarding recommended treatment dosage:

*“I gave him the medicine*. *But not the full dose*. *He had no money for the combination treatment so I gave him one drug that would help him for the problem*.*”*

(Informant 10, female)

Statements also indicated an inclination of ‘*financial incentives superseding need for prescription’*:

*“As long as they can pay for the drugs and have told me what problems they suffer from I feel I can give it to them*.*”*

(Informant 2, female)

As demonstrated by the quote, the interest to make money was embedded in a rationale that if informants could describe their symptoms, assistance was to be provided.

*‘Establishing a good reputation in MSM community’* seemed to be fundamental to reach and retain clients. Pharmacy workers considered the confidential aspects of their work to be crucial for building credibility and reaching out to MSM.

*“I have been told that many people say to others ‘That lady in* [name of location, author’s remark], *she is good*, *she keeps secrets’*. *So many comes here after hearing about me from their friends*.*”*

(Informant 3, female)

This reputation was considered to be a facilitator for clients to come forward, sometimes “*returning with another one* [MSM, author’s remark]” (Informant 1, female).

### Being challenged by others for what I do

The transition into a regular service provider for MSM clients was complicated by the surrounding environment’s reactions against homosexuality. Efforts to provide adequate services to MSM clients were hampered by a complex interplay of society’s limited knowledge of and education about homosexuality, existing socio-cultural norms, and negative attitudes towards helping MSM.

*‘Colleagues objecting to assist MSM clients’* appeared to be commonly encountered among pharmacy workers. Informants explained that the reactions stemmed from the negative attitudes some colleagues held against MSM clients and their work:

“*Some of my colleagues they do not even want to see that type of people here and they do not agree with what I am doing*.”

(Informant 2, female)

Informants characterised these colleagues as having “*a lack of courage*” (Informant 7, female). However, they also reasoned around the invisibility of homosexuality during their professional training, e.g. “*it is like we never talked about it* [homosexuality, author’s remark] *at college*” (Informant 7, female), and the impact of this on pharmacy workers’ ability to deal with MSM clients, e.g. “*you are not being prepared for all this when you studying*” (Informant 1, female).

When describing their work with MSM clients, ‘*community reacting against me’* emerged as a consequence. Informants’ accounts suggested that they were stigmatised as they became associated with their clients:

*“If a MSM comes by and greet me or talks to me the community starts pointing fingers and say ‘She is also doing anal sex’*. *The community has created something around me*. *So stigma is a problem*.*”*

(Informant 16, female)

Informants also felt accused when community members questioned their intentions:

*“So when you are helping MSM they* [community members, author’s remark] *ask ‘Why are you helping these people*? *Are you one of them*? *What do you want*? *You want them to take your husband*?*’*. *There are many people who think they* [MSM, author’s remark] *want to take their families*.*”*

(Informant 7, female)

*‘Coping with accusations’* occurred in different ways, for example by referring to kinship “*what if it is your child or your brother*?*”* (Informant 8, male) or with irony:

*“They could say ‘So you want him to take your husband?’ I say ‘Yes, there is no problem’*.*”*

(Informant 7, female)

Irony was not only used as a counter strategy against accusations but also to “*hide that you are hurt*” (Informant 7, female).

Accusations, however, spurred frustration. As illustrated by the following statement, informants were challenged by the questions from community members:

*“Some ask me ‘Why do you provide services to them? Leave them to die, leave them to die’, and I answer that they are human beings*. *I do not get sad*. *But it disturbs me*. *Why cannot these people let me do my job?”*

(Informant 12, female)

## Discussion

The main finding suggests that pharmacy workers’ involvement with MSM clients was the result of a gradual process labelled “*Stretching the boundaries for pharmaceutical responsibilities”*, the core category (Fig 2). ”Stretching”, defined as”the act of expanding by lengthening or widening” [26] the boundaries was chosen as a metaphor for the process identified in our data. This alludes to stepping out of the comfort zone both on a personal and a professional level in order to cater to a vulnerable group (MSM) in dire need of medical services. Circumventing culturally, socially, and religiously accepted values was required in order to accomplish this, as well as extending the limits of own professional duty assignments, all of which will be discussed below in relation to the categories highlighting these findings. In the field of nursing, Torn and McNichol used the metaphor “stretching boundaries” to refer to nurses’ professional risk-taking, perceived as a professional maturity allowing certain flexibility to established rules [27]. Challenging established rules and regulations based on critical knowledge gained from own professional experience has also been described by Patricia Benner in her research on the expert nurse [28, 29]. This may be relevant to the findings of our study, as the experience of catering to MSM clients led to new approaches to service provision, which subsequently laid the foundation for becoming regular service providers to the MSM population. At the same time, dispensing drugs without a prescription and thereby assuming the role of medical doctor suggests that there were challenges related to acknowledging own professional limitations.

### Developing interpersonal contacts

Pre-existing knowledge and attitudes about homosexuality appeared to influence how clients were evaluated as reflected in the category *Recognising the MSM client* (Fig 2a). Yet, the involvement with clients seemed to have resulted in the formation of positive ties. This suggests that interpersonal contacts had a beneficial effect on the development of positive attitudes toward the stigmatised group, in accordance with Allport’s contact hypothesis [30]. However, few studies in sub-Saharan Africa have examined the conditions under which positive attitudes towards homosexuality develop, and additional studies would be required to determine the role of interpersonal contact and its relation to other factors.

### Searching in religion to find answers

The category *Arriving at acceptance through gradual exposure* (Fig 2c) addressed the process of becoming susceptible to MSM clients’ needs. An interesting observation is that religion seemed to play a role in the process, as religious precepts regarding equality were called to mind. This is in contrast to other studies on homosexuality and MSM in Sub-Saharan Africa, where homophobia has been attributed to religious influence [31–34]. However, even our findings disclosed that conflicts and tensions in reconciling faith with homosexuality were a reality. It is possible that religion was important for developing responsiveness to clients’ needs in that it gave a sense of meaning to efforts to assist clients but without necessarily requiring that pharmacy workers *accepted* homosexuality.

### Transgressing boundaries

As illuminated in the categories *Becoming aware of MSM clients’ predicaments and Motivation to help clients driven by compassion* (Fig 2b and 2d), the process of pharmacy workers’ engaging with their MSM clients resulted in new approaches to service provision for these clients. As commercial actors pharmacy workers are required to be helpful towards the general community in order to retain customers. In low-resource settings, where healthcare access and ability to pay for medical consultation are limited, this implies duties beyond only prescribing drugs, as pharmacists widely are used also as health advisors [35–37]. This agrees with our findings but we also discerned that the personalised ties with MSM clients that had emerged appeared to trigger pharmacy workers to adjust their services to better meet the needs of these clients. As pharmacy workers began to understand the situation that MSM clients faced with regards to stigma and discrimination, they became increasingly aware of clients’ emotional needs. Thus, they increasingly extended services beyond providing drugs to also focussing on counselling and motivation–aspects that are in keeping with the pharmacist’s professional role.

However, findings also revealed that these seemingly altruistically motivated gestures sometimes blurred provider-client boundaries, which consequently were transcended. There is a rich body of literature in the medical and sociological fields regarding boundary transgression in healthcare [38–41]. When a practitioner develops an emotional commitment to a patient or client, the decision-making concerning care and treatment might be threatened as the practitioner’s ability to be objective is compromised.

In this study pharmacy workers were aware of the double burden MSM clients faced with regards to STI problems and stigma in healthcare. Despite the belief that clients would be left without treatment otherwise, the administration of drugs without a prescription raises some concerns about “professionalism”, i.e. adherence to regulations regarding pharmaceutical services. Nevertheless, emotional commitment to clients might have created difficulties regarding the separation of the professional role from the non-professional role in decision-making situations. The Tanzanian government’s efforts to transform local drugstores, so called *Duka la Dawa Baridi* (Part II pharmacies), into government-accredited drug dispensing outlets (also called the ADDO programme) is a step towards increasing professionalism by sensitising staff on best practices for treatment [21, 42]. Such training should also attempt to increase pharmacy workers’ factual knowledge of STI treatment guidelines to address knowledge gaps leading to incorrect drugs, dosages and duration, as found in previous studies [43–45].

### Identifying financial interests

The identification of financial motives in service delivery as described in the category *Acting on the basis of own financial interests* (Fig 2e) is an important finding of this study. It indicates that commercial motives may have played a role in the willingness to respond to clients’ needs, which contributed to insufficient drug dosages for clients’ STI symptoms. From a profit-making perspective, providing drugs for free could threaten the sustainability of a business. This finding underscores the dilemma related to ethics and profit-making enterprises in the health sector, which inevitably could have effects on the quality of STI treatment and management.

The “double role” of pharmacy workers as healthcare providers and businessmen has also been reiterated in other studies from low-income countries [7, 46, 47]. A study from Hanoi, Vietnam, revealed how pharmacists attempted to maximise profits by promoting certain drugs (i.e. Ampicillin) that were considered to generate the highest profit [47]. While there might be incentives for a provider to promote him or herself in the community as someone who helps people and not only provide “quick-fixes” [7], our findings suggest that financial aspects compromised accurate STI management. Over- and misuse of antibiotics have been attributed to antimicrobial susceptibility at a global level [48], and an increasing trend of drug resistance has been observed in Tanzania [49]. The attempt to support pharmacy workers to make joint procurements of drugs to reduce unit costs, as outlined in the ADDO programme [42], could be a step towards decreasing expenditures and aiding them in achieving financial sustainability. In the wider perspective this may contribute to reduce financial incentives related to unsupervised drug provision.

Mugo et al. in a study on STI treatment and HIV referral practices among Kenyan health providers found that only 10% of pharmacists recommended HIV-testing to their clients in simulated visits [43]. However, the authors highlight the potential represented by utilising pharmacists to extend HIV-testing and referral for ART treatment to clients, who seek their services for STI symptoms. This is relevant also in the context of the present study since pharmacy workers may be the only contact that MSM clients have with the healthcare system due to stigma and discrimination in clinics and hospitals. Such a suggestion is well aligned with current Tanzanian HIV guidelines, which stress that MSM are still a population at very high risk and are therefore crucial to reach for HIV screening and treatment [50]. By providing training in HIV-testing and counselling, pharmacy workers could potentially play an important role in linking HIV positive MSM with treatment services.

### The role of secondary stigma

Pharmacy workers were confronted by colleagues and community members regarding the services provided to MSM clients as reflected in the category *Being challenged by others for what I do* (Fig 2f). Herek and colleagues, among others, have discussed the role of secondary stigma in the area of HIV and AIDS [51]. This phenomenon occurs when stigma is directed toward those who are not infected by HIV but in various capacities are related to a HIV infected person. Our findings imply that pharmacy workers were targets of secondary stigma as they became associated with their MSM clients. In order to encourage continued engagement with MSM clients, it is imperative to support pharmacy workers in coping with the stress and anxiety that secondary stigma may result in.

### Methodological considerations

The study provides valuable information about pharmacy workers’ experience of providing STI services to MSM clients. Several measures were taken to consolidate the study’s trustworthiness. Prior to data collection, the first author (ML) and the last author (AA) discussed the sexual health needs of MSM with key informants from the MSM community and the healthcare sector, to gain an understanding of the culture and social setting. Furthermore, to enhance the credibility of the findings, the preliminary analysis was discussed with key informants from the MSM community. [52]. Dependability and confirmability were assured through documentation of each step of the study, and by providing detailed quotations that showed how findings had been interpreted [52]. This study was localised to selected pharmacies and drug stores in the greater Dar es Salaam area and the findings are unique for the context in which they occurred. This raises important questions regarding the transferability of the findings [52]. Albeit qualitative studies do not attempt to generalise findings, their description of a phenomenon could be valid in another setting, i.e. the transferability. Given the human and financial constraints that characterise the health sector in sub-Saharan Africa [53] and persistent stigma against same-sex sexuality [54, 55], it is possible to assume that the findings are relevant also in the wider geographical setting.

The study also had some limitations. Since we purposively selected pharmacy workers who were already engaged in MSM care, pharmacy workers who might oppose such engagement were not included. Furthermore, the gender imbalance in the sample, three males and thirteen females, might influence the transferability of the findings to other settings. Although global and regional data suggest an increasing number of women in the pharmacy profession, the proportion of males remains higher [56, 57]. An explanation for the imbalance in our sample probably stems from the recruitment strategy, as we used an inventory list of pharmacy workers, provided by MSM members of our local partner organisation. Hence, the gender imbalance might be a reflection of provider preferences among MSM clients, and should be taken into consideration when transferring the findings to other settings.

The language barriers faced in this study constitute another limitation. Even if English is widely spoken in Tanzania, Swahili is the official language and mother tongue of most people. To address potential language barriers it was considered important to use an interpreter to provide simultaneous translation when necessary. Although we recruited a person from the MSM community to ensure that he had an in-depth understanding of the topics discussed, it is possible that some words and underlying meanings were lost in the process of translation. To reduce errors, the first author listened to the recordings together with the interpreter after the interview to get a second translation, and compared this with his notes from the interview situation to ensure that they corresponded. The role of the interviewer also raises important questions since both interviewers were non-Tanzanian and outsiders to the particular context of pharmacy workers. Hence, there is a possibility that they failed to understand the depth and breadth of the lived experiences of the informants, which potentially could have influenced the data collection process and assessment of data saturation. To mitigate such risks the two investigators kept analytical memos that summarised questions, findings, and impressions from the interviews. These were shared and discussed among the authors in order to reveal and contest own pre-conceptions and biases.

## Conclusions and Recommendations

Pharmacy workers play an essential role for stigmatised populations such as MSM since they may be one of the very few contacts that these men have with the healthcare system due to fears of stigma and discrimination. This study provides a greater understanding of the factors that influence pharmacy workers’ involvement in MSM clients and their efforts to provide treatment for STIs. However, the unsupervised sale of drugs could affect quality of treatment and contribute to further drug resistance development. More studies are required to examine how pharmacy workers arrive at their choices in light of existing guidelines and protocols. A concerted effort on the part of governmental authorities is also required to address the over- and misuse of antibiotics. Such initiatives need to take into account the financial interests potentially underlying inaccurate or incomplete drug provision. However, pharmacy workers currently remain an untapped resource for extending HIV-testing to MSM given their access to this hard to reach population. Government authorities should consider the possibility to train pharmacy workers to carry out HIV-testing and provide referrals for MSM clients, thereby making them frontline workers in the HIV care continuum. Finally, inter-professional networks between pharmacy and healthcare workers could be influential in supporting the development of knowledge exchange on key populations and referral systems for diagnosis, care, and treatment.

## Supporting Information

S1 File Interview guide (English). (DOCX)

S2 File Interview guide (Kiswahili). (DOCX)
