# Supplementary material for: Stretching the Boundaries: Tanzanian Pharmacy Workers’ Views and Experiences of Providing STI Services for Men Who Have Sex with Men
Source: PLoS One. 2016 Nov 3;11(11):e0166019. doi: 10.1371/journal.pone.0166019 (PMC5094583; doi:10.1371/journal.pone.0166019)
Supplement: S1 File — (DOCX) [file pone.0166019.s001.docx]

**Thematic Interview Guide**

**Consent form- read aloud**

My name is ____. We are currently conducting a study on the views and opinions of providing services related to sexually transmitted infections to men who have sex with men among people working at pharmacies and drugstores in Dar es Salaam. The findings of the study will contribute to an improved understanding of health care provision to perspective sexual minorities from a pharmaceutical perspective and also provide important information on how to more effectively access and engage men who have sex with men in health care provision.

The study is co-coordinated by the University of Dar es Salaam, Muhimbili University of Health and Allied Sciences and Lund University (Sweden).

Your participation in the study is completely voluntary, you will not be forced to answer questions that you do not want to answer and you may end the interview at any time you want to. We would also like to assure you that all information collected in the course of the study will remain confidential. Your name will not be written on the interview scripts in a manner that someone can identify the source of information. As you will be anonymous, there are no risks associated with participation in this study.

However, this information may be used in scientific publications or presentations to increase the awareness of HIV/AIDS and STIs among men who have sex with men. Your information will be completely anonymized and no one will be able to trace your answers back to you.

The interview will take approximately one hour.

In case you have any questions, concerns or comments related to this study feel free to contact the following people who will provide you with the necessary assistance (provide card with contact information).

Do you have any questions?

**Do you agree to participate in this interview? Say “yes” or “no” and today’s date.**

**Opening question:**

Would you like to tell me about your workplace?

*Probes:*

*How long have you worked here?*

*How many work here?*

*Your educational background?*

**Services**

In Tanzania, as well as in other countries, syndromic management is used to diagnose STI:s. Would you like to tell me about your experience of this?

*Probe:*

*Could you tell me what types of clients that come to this pharmacy for STIs? Both men and women?*

As you know this study intends to focus on male clients who have sex with other men (MSM)- I wonder if you would like to tell me about your experience of this clientele?

Would you like to tell me how these clients approach you? You are an authority but they are in need of your services?

In your experience, what are they willing to disclose when they approach you?

*Sentiments:*

*Are they shy?*

*Are they afraid?*

*Are they comfortable?*

*Probe:*

*How did you manage to get them to trust you?*

What kind of problems to the MSM clients bring to you?

*Probe:*

*Type of STI*

What services do you provide here when it comes to STI:s?

*Probe:*

*Do they come during certain hours?*

*Who attends them? Who do they ask for to attend them?*

*Possibilities to privacy?*

You have explained your services here but are there other types of services that are provided here to MSM?

**Challenges**

What do you feel are the challenges of providing services to this clientele?

*Sentiments:*

*Attitudes?*

*Dislike?*

*Despise?*

*Religious doubts?*

*Probe:*

*What kind of support do you receive for providing these services?*

We have talked about challenges here, how could services at pharmacies be improved to ensure that this clientele receives services?
