# Supplementary material for: Stretching the Boundaries: Tanzanian Pharmacy Workers’ Views and Experiences of Providing STI Services for Men Who Have Sex with Men
Source: PLoS One. 2016 Nov 3;11(11):e0166019. doi: 10.1371/journal.pone.0166019 (PMC5094583; doi:10.1371/journal.pone.0166019)
Supplement: S2 File — (DOCX) [file pone.0166019.s002.docx]

**Muongozo wa mahojiano**

**Fomu ya ridhaa – Soma kwa sauti**

Jina langu ni____. Tunafanya utafiti kujua mtazamo na maoni ya wafanyakazi wa famasi na maduka ya dawa muhimu juu ya utoaji huduma za magonjwa ya zinaa kwa wasenge (Wanaume wanaofanya mapenzi na wanaume) hapa Dar es Salaam. Matokeo ya utafiti huu yatasaidia katika kuongeza uelewa juu ya utoaji huduma za afya kwa mashoga hasa katika uuzaji na utoaji dawa na pia yatatoa taarifa muhimu juu ya jinsi ya kuwafikia na kuwashirikisha wanaume wanaofanya mapenzi na wanaume wenzao katika utoaji wa huduma za afya.

Utafiti huu unaratibiwa kwa ushirikiano wa Chuo kikuu cha Dar es Salaam – *Chuo kikuu cha Afya na Sayansi shirikishi cha Muhimbili* na Chuo kikuu cha Lund (Sweden).

Ushiriki wako katika utafiti huu ni wa hiari, haulazimishwi kujibu swali ambalo hauko tayari kujibu na unaweza kumaliza au kuahirisha mahojiano muda wowote utakaohitaji kufanya hivyo. Pia tunakuhakikishia kwamba taarifa zote zitakzokusanywa wakati wa utafiti huu zitabaki kuwa siri. Jina lako halitaandikwa katika namna ambayo mtu anaweza kubaini chanzo cha taarifa. Hautajulikana na hakuna hatari yoyote kwa wewe kushiriki utafiti huu.

Hata hivyo, taarifa hizi zinaweza kutumika katika machapisho au mihadhara mbalimbali ya kuongeza uelewa juu ya VVU/UKIMWI na magonjwa ya zinaa miongoni mwa wanaume wanaofanya mapenzi na wanaume. Taarifa zako zitafichwa chanzo chake na hakuna atakayefuatilia taarifa hizi kwako.

Mahojiano yatachukua saa moja.

Kama una swali,dukuduku au maoni juu ya utafiti huu jisikie huru kuwasiliana na watu wafuatao ambao watakuwa tayari kutoa msaada unao hitajika (Toa kadi yenye taarifa za mawasiliano).

Je una swali lolote?

**Je,unakubali kushiriki mahojiano haya? Sema “Ndiyo” au “Hapana” na tarehe ya leo.**

**Swali la ufunguzi:**

Unaweza kutueleza kuhusu kituo chako cha kazi?

*Dadisi:*

*Umekuwa hapa kwa muda gani?*

*Watu wangapi wamefanya kazi hapa?*

**Huduma**

Je unaweza kutueleza uzoefu wako kuhusu usimamizi wa dalili katika kutambua magonjwa ya ngono ukizingatia Tanzania ni moja ya nchi zinazofuata mfumo huo (Syndromic Management).?

*Dadisi:*

*Je unaweza kunieleza ni aina gani ya wateja wanaokuja kwenye duka lako la madawa kwa ajili ya magonjwa ya ngono?? Wake kwa waume?*

Kama unavyofahamu tafiti hii inakusudia Zaidi kwa wanaume (Kuchu), Tafadhali waweza nielezea chochote kuhusu kundi(Kuchu/ MSM) hili?

Tafadhali unaweza nielezea namna Kuchu/ MSM wanavyokufikia/ kuuliza? Wewe kama mwenye duka lakini pia kupata huduma

Kwa uzoefu wako , ni vitu gani wanavyokueleza wanapokuja kupata Huduma?

*Hisia*

*Wanaona aibu?*

*Wanaogopa*

*Wako huru?*

*Dadisi*

*Uliwezaje kuwafanya wakuamini?*

What kind of problems to the MSM clients bring to you?

*Dadisi:*

*Aina ya magonjwa ya ngono*

Ni aina gani ya Huduma unayotoa hapa linapokuja suala la magonjwa ya ngono?

*Dadisi:*

*Je kuna muda maalumu ambao wanakuja?*

*Nani anawahudumia?*

*Nani wanamuuliza kwa ajili ya kuwahudumia?*

*Suala la Usiri?*

Umesahanieleza kuhusu Huduma unazotoa hapa , lakini kuna Huduma nyingine za ziada unazotoa hapa kwa Kuchu/ MSM?

**Changamoto**

Unadhani ni changamoto zipi kwa kutoa Huduma kwa Kuchu/ MSM?

*Hisia:*

*Mtazamo?*

*Usichopenda?*

*Unacopenda?*

*Mtazamo wa kidini?*

*Dadisi:*

*Aina gani ya msaada unayopata hapa kwa kutoa hizi Huduma?*

Tumezungumzia kuhusu changamoto, unaonaje kuhusu fursa katika kuhudumia kundi hili??
